# Gender and line size factors modulate the deviations of the subjective visual vertical induced by head tilt

**DOI:** 10.1186/1471-2202-13-28

**Published:** 2012-03-15

**Authors:** Marion Luyat, Myriam Noël, Vincent Thery, Edouard Gentaz

**Affiliations:** 1 Department of Psychology, University of Lille, Laboratory of Functional Neurosciences and Pathology EA4559, 4 rue du Barreau, Villeneuve d'Ascq, 59653, France; 2 Service MPR Neurologique, Centre Hospitalier de Saint-Amand-les-Eaux, 19 rue des Anciens d'A.F.N., Saint-Amand-les-Eaux, 59230, France; 3 Department of Psychology, University of Grenoble, Laboratory of Psychology and NeuroCognition UMR 5105, 1251 avenue centrale, 38040, France

## Abstract

**Background:**

The subjective visual vertical (SVV, the visual estimation of gravitational direction) is commonly considered as an indicator of the sense of orientation. The present study examined the impact of two methodological factors (the angle size of the stimulus and the participant's gender) on deviations of the SVV caused by head tilt. Forty healthy participants (20 men and 20 women) were asked to make visual vertical adjustments of a light bar with their head held vertically or roll-tilted by 30° to the left or to the right. Line angle sizes of 0.95° and 18.92° were presented.

**Results:**

The SVV tended to move in the direction of head tilt in women but away from the direction of head tilt in men. Moreover, the head-tilt effect was also modulated by the stimulus' angle size. The large angle size led to deviations in the direction of head-tilt, whereas the small angle size had the opposite effect.

**Conclusions:**

Our results showed that gender and line angle size have an impact on the evaluation of the SVV. These findings must be taken into account in the growing body of research that uses the SVV paradigm in disease settings. Moreover, this methodological issue may explain (at least in part) the discrepancies found in the literature on the head-tilt effect.

## Background

On Earth, humans need to have a sense of verticality. In sensorimotor terms, our upright, bipedal, postural stance is mediated by vestibular, somesthetic and visual inputs that serve as indicators of any deviation from the vertical. On the cognitive level, our vertical perception defines a gravitational reference frame, which subserves the coding of the location and orientation of objects in the environment independently of the observer's own orientation. Consequently, the subjective vertical (SV, i.e. the subjective estimation of gravitational direction) is commonly considered as an indicator of the sense of orientation. The SV is measured by asking the observer to align a light bar with the direction of gravity (i.e. the subjective visual vertical, SVV). Other modalities (such as the haptic modality) have been also used (e.g. [1-9]).

The SVV arises from the complex integration of inputs from vestibular, visual, proprioceptive and tactile receptors. It has been clearly established that subcortical structures are involved in the vestibular contribution to oculomotor control (vestibulo-oculomotor reflexes) and postural control (vestibulo-collic and vestibulo-spinal reflexes) (for a review, see [10]). However, it is not known precisely how and where in the cortex the vestibular information on spatial cognition (and on the sense of verticality, in particular) is processed. Otoliths and semicircular canals give rise to vestibular inputs, which run from the eighth nerve to the vestibular nuclei at the pontine level. Studies in monkeys [11] have shown that after thalamic projection, the signals reach (directly or indirectly) several areas of the vestibular cortex (areas 2v, 3a and 7, in particular), the parieto-insular vestibular cortex (PIVC) and the ventral intraparietal area. Functional MRI studies [12,13] have revealed that similar areas are involved in humans (particularly the PIVC and temporal areas), with right-hemisphere dominance. According to Barra et al. [14], the representation of verticality may depend on neural circuits that include thalamoparietal projections (for somesthetic graviception) and thalamo-insular projections (for vestibular graviception).

Lesions or impairment at any of the steps in vestibular information processing can induce pathological deviations of the SVV or the subjective visual horizontal. Indeed, this paradigm has frequently been used to detect acute, unilateral vestibular defects in disease settings [15]. However, thalamic infarction [16] and cortical damage (especially in parietal areas and, more specifically, damage to the PIVC) can also induce deviation of the SVV (for a review, see [17]). For example, in the study by Brandt et al. [18], contraversive tilts of the SVV were found in 33 out of 52 patients with brain damage in the PIVC area; ocular torsion was ruled out as a possible cause of the deviation. Most studies on stroke patients have reported an altered sense of verticality and a subjective vertical tilted towards the contralesional side - especially in patients suffering from hemineglect syndrome [19-21]. Nowadays, a growing number of researchers use the SVV paradigm to investigate other diseases, such as paraplegia [14] and psychiatric conditions [3].

However, even healthy observers will suffer from non-negligible, biased accuracy if visual cues are not available and the head axis is no longer relevant (when roll-tilted, for example). Many literature reports show that (i) head tilts of up to 60° can give rise to contraversive displacement of the SVV (the Müller effect, also known as the E-effect) and (ii) greater tilt angles induce systematic deviations in the head tilt direction (the Aubert effect, also known as the A-effect). Nevertheless, a review of the literature on head-tilt effects as a function of the amplitude of head tilt reveals strong disagreements with respect to the specific values and conditions that yield A- or E-effects. For example, several researchers have found systematic A-effects with moderate head tilts of about 30° [2,22-25], whereas others always observed an E-effect under similar conditions [26,27]. Furthermore, some researchers report high, between-subject variability rather than an average, systematic effect of head tilt [28-30]. Table 1 provides a summary of these various experiments and their main results.

**Table 1:** Results obtained in different studies on the Subjective Visual Vertical (SVV) or Subjective Visual Horizontal (SVH).

Authors	Sample	Physical size	Distance	Angular size	Orientation	Tilt	Results
Wade (1969)	10 subjects, sex no specified (ns)	91.5 cm et 15.5 cm (2.1 cm wide)	180 cm	28.52° et 4.93°	SVV	± 30° (Head Tilt)	E-effect on average Decrease of the E-effect with large bar

De Graaf et al. (1992)	7 men, 5 women (age between 19-32 yr)	50 cm	125 cm	22.6°	SVH	0°, ± 5°, ± 10°, ± 15°, ± 20°, ± 25° (Body Tilt)	On average, no deviation from upright. 5 subjects had E-effect, 3 others an A-effect, the remaining 4 subjects were not easily determined: 3 of them tended to show an E-effect and 1 tended to show an A- effect

Tribukait et al. (1996)	39 men and 36 women (37 ans), 3 were excluded but their sex was not specified	ns	ns	6.5°	SVH	0°, ± 10°, ± 20°, ± 30 (Body Tilt)	High between-subjects variability but E-effect on average

Guerraz et al. (1998)	35 women and 34 men (age between 18 and 22 yr)	21 cm	ns	20°	SVV	± 28° (Head Tilt)	A-effect

Van Beuzekom & Van Gisbergen (2000)	5 men and 1 woman (age between 20 and 54 yr)	ns	100 cm	17°	SVV and SVH	Between -180° and +180° with a 10° interval (Body Tilt)	High between-subjects variability, for small angle of tilt, some subjects show E-effects, others, A-effects

Guerraz et al. (2000)	N = 20 sex no specified (age between 18 and 22 yr)	21 cm	60 cm	19.85°	SVV	0° ± 7, ± 14, ± 21, ± 28, ± 35° (Head tilt)	A-effect on average from 7° to 35° of head-tilt

Mast (2000)	1 man and 3 women (age between 26 and 32 yr)	ns	120 cm	8°	SVV	Angles between 0° and 180° at each 15° intervals 0°, 15°, 30, 45°...180° (Body Tilt)	A small (no precise value) A-effect for tilt angle up to 135°

Luyat & Gentaz (2002)	7 women and 2 men (mean age = 22.4 yr)	27 cm	68 cm	22.46°	SVV	± 45°	A-effect

Trousselard et al. (2003)	5 women and 11 men (mean age: 28 yr)	32 cm long (1.5 cm wide)	55 cm	32.44°	SVV	0°, ± 15°, ± 30°, ± 45°, ± 60°, ± 75°, ± 90°, ± 105° (Body Tilt)	A-effect

Kaptein & Van Gisbergen (2004)	6 men (age between 23-59 yr)	ns	90 cm	20°	SVV	Angles between 0° and 360° at each 30° intervals 0°, 30, 60°, 90°...360° (Body Tilt)	Limited time of adjustment (30 sec) For angle ≤ 60°: 2/6: E-effect, 4 gave veridical response or small A-effect (no precise value)

ns = no-specified

In fact, if we consider moderate tilts and normal subjects, two main factors appear to vary strongly from one study to another (see Table 1): the angle size and the participants' gender (when this information is mentioned). In experiments showing a systematic A-effect [2,22-25], very large stimuli were used (from 19.85° to 32.44°). Although the effect of the stimulus angle size was tested some time ago by Wade [26], it merits renewed investigation. In fact, Wade found that a large angle size could diminish the E-effect but did not find an A-effect - even with a large angle size (28.52°). Unfortunately, data concerning other aspects of study design (such as whether participants of both genders or just one gender were included) were not specified.

Several spatial tasks show gender effects, although the latter are not completely understood. Anatomical explanations have been proposed [31,32]. The otolithic organs (i.e. the utricle and saccule) and the superior semicircular canals appear to be larger in men than in women [33]; this may explain (at least in part) gender-related differences in vestibular information processing (see [31,32]). Since the pioneering work by Asch and Witkin [34], it has been well documented that women are usually more affected by visual, contextual cues (such as those used as in the rod-and-frame test and the water-level task, for example [35,36]). Gender effects are also frequently mentioned in spatial attention tasks, with poorer performance levels by women (although a correlation with the functional differences revealed by fMRI has not been found [37]). In navigation tasks, significant differences between men and women have also been evidenced [38]. However, gender differences in spatial tasks do not appear to be limited to paradigms involving visual contextual cues, since Tremblay et al. [31,32] found gender differences in judgment of the morphological horizon in different body orientations. A few studies have sought to identify a gender difference for the SVV during head-tilt (and in the absence of visual context) [36,39] but failed to do so.

In order to explain some of discrepancies concerning head-tilt effects for the SVV, we decided to investigate the possible impact of methodological factors (the line's angle size and the participant's gender) with a moderate (30°) head tilt. This question is important in view of the growing body of research using the SVV paradigm in various disease settings. Forty healthy participants (20 men and 20 women) were asked to make visual vertical adjustments of a light bar with their head positioned vertically or roll-tilted by 30° to the left or the right. Line angle sizes of 0.95° and 18.92° were presented.

## Method

### Participants

The 40 study participants (20 women (mean ± SD age: 23.20 ± 3.22) and 20 men (mean ± SD age: 24.10 ± 3.04)) were all psychology or neuroscience students. A T-test failed to show any difference between the gender groups' respective mean ages (t_38 _= -0.90; *p *= 0.37). The participants' vision was normal or corrected-to-normal with lenses. According to self-reports, none of the subjects had hearing or vestibular disorders, diseases with ocular effects or motion sickness. The subjects were all volunteers and informed consent was obtained after a full explanation of the experimental procedure. In France, our behavioral, non-invasive, non-medical study did not require approval by an independent ethics committee. However, we did follow the recommendations of the French Psychology Society http://www.sfpsy.org/spip.php?rubrique27.

### Materials

**Figure 1:**
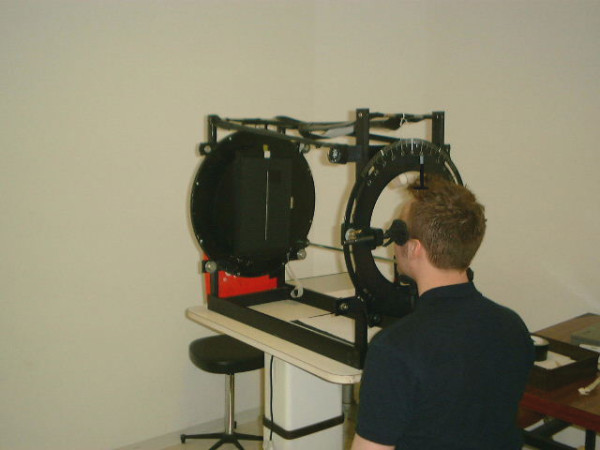
**The experimental apparatus**. During the experiment, the whole apparatus was covered by a black cloth (to prevent contextual visual cues).

The equipment used to present the stimulus comprised of a 60-cm-long tunnel in a metal framework (see Figure 1), which was covered by a black cloth during the experiment to block any visual, contextual cues. At the bottom of this device was a rotating metal disc (diameter: 44 cm) fitted with electroluminescent diodes. A rigid black plate with an adjustable aperture (corresponding to the line size) was placed in front of the diodes to provide either a small angle size for the emitted light (angle: 0.95°; length: 1 cm; width: 1 mm) or a large angle size (18.92°; length: 20 cm; width: 1 mm). The disc could be rotated by up to 240° around its central axis. The display was viewed binocularly. The back of the disc was graduated in degrees and the display's sensitivity threshold was ± 0.5°.

At the aperture of the visual apparatus, the participant's head was held in place at four points (the temples, the top of the head and the chin placed on a padded rest), which allowed the head to be held upright or tilted by 30° to the left or to the right. The participant was seated in front of the visual apparatus at an appropriate height, with his/her head placed in the head rest device.

### Experimental conditions and procedures

The participants had to adjust the rod to the gravitational vertical as accurately as possible. No time limit was imposed. A gravitational definition of the vertical was given to the participants (the direction of a plumb line; a concrete example was shown). The two chosen light bar angles (0.95° and 18.92°) were presented in separate sessions, with a 5 min interval. For each angle size condition, the participant adjusted the line in three head posture conditions: (i) the head held vertically (the baseline condition), (ii) the head roll-tilted to the left (- 30°, by convention) and (iii) the head roll tilted to the right (+30°). The baseline condition was performed first and the order of presentation of the left-tilt and right-tilt conditions was randomized. In each of these six experimental conditions (two angles × three head positions), there were four adjustments to the vertical. The initial rod position was 20° away from the physical vertical and the direction of displacement (clockwise and counter-clockwise) was counterbalanced over the four trials.

## Results

The results are depicted in Table 2.

**Table 2:** The visual subjective vertical in the three head conditions as a function of angular size and gender.

	Head to the Vertical		Head Tilted to the Left		Head Tilted to the Right	
	**Angular size of the luminous bar**					

Gender	0.95°	18.92°	0.95°	18.92°	0.95°	18.92°

Women	-0.394 (0.304)	-0.619 (0.245)	-1.022 (0.731)	-2.500 (0.605)	-0.666 (0.640)	0.503 (0.545)

	Min = -3.375	Min = -2.25	Min = -9.5	Min = -8.25	Min = -10.5	Min = -3.75

	Max = 3.4375	Max = 2.125	Max = 2.875	Max = 1.875	Max = 1.875	Max = 5.5

Men	-1 (0.304)	-1.222 (0.245)	0.744 (0.731)	-0.972 (0.605)	-2.131 (0.640)	-1.334 (0.544)

	Min = -3.437	Min = -2.25	Min = -9.5	Min = -8.25	Min = -10.5	Min = -3.75

	Max = 0.875	Max = 0.375	Max = 7.75	Max = 3.375	Max = 1.5	Max = 4.5

Standard errors of the mean are noted in parentheses (measures expressed in degrees)

For each trial, the deviation (in degrees) of the line's final position from the gravitational vertical was first noted. By convention, deviations to the left (i.e. with the rod turned counter-clockwise with respect to the gravitational vertical, as viewed by the subject) were counted as negative and deviations to the right (clockwise) were counted as positive. The mean of the algebraic deviations (in degrees) over the four trials in each postural condition was computed. The threshold for statistical significance (i.e. for rejection of the null hypothesis) was set to p < 0.05 for all tests.

**Figure 2:**
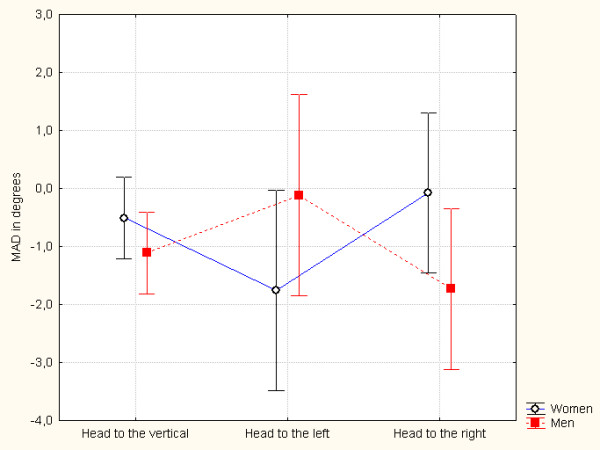
**The subjective visual vertical (as the mean of algebraic deviations (MAD), in degrees) in women and in men as a function of the head position**. The vertical bars represent the 95% confidence interval.

The standard assumptions for performing an analysis of variance (ANOVA) were validated, i.e. the normality of the distributions (checked with a Kolmogorov-Smirnov test) and the homogeneity of the variance matrix between groups (checked with Box's M test; *p *> 0.40). We performed a two (*gender*) × three (*head position*) × two (*angle size*) factor ANOVA with repeated measures on the latter two factors. This analysis did not reveal a significant effect of *gender *(F_(1,38) _= 0.344; *p *= 0.56) or *head position*(F_(2,76) _= 0.038; *p *= 0.963). In contrast, the interaction between *head position *and *gender *was statistically significant (F_(2,76) _= 5.960; *p *= 0.0003 (see Figure 2)). However, an orthogonal contrast analysis revealed a significant difference between women and men in the "right-tilt" condition (F_(1,38) = _5.866; *p *= 0.02). In the other head positions, the effects showed a trend but failed to achieve statistical significance (F_(1,38) _= 3.069, *p *= 0.08, for the "head vertical" condition; F_(1,38) _= 3.721, *p *= 0.06 for "left-tilt").

Even though the mean size of the deviations was moderate (with high between-subject variability; see Table 2), the effect of the head-tilt differed according to *gender*. In women, a deviation in the direction of head tilt (i.e. an A-effect) was observed (vs. the baseline, "head vertical" condition), whereas the opposite was found in men (i.e. an E-effect).

**Figure 3:**
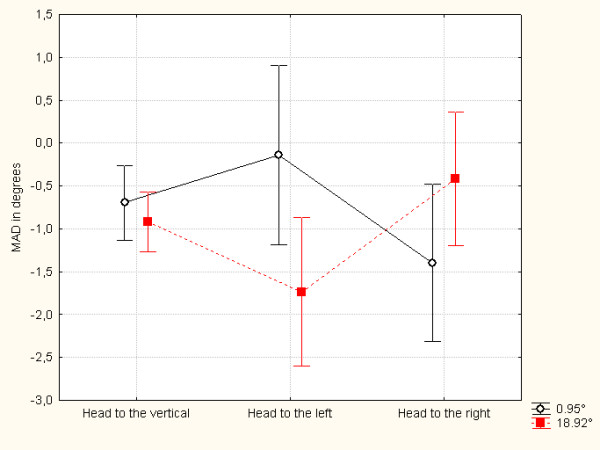
The subjective visual vertical (as the mean of algebraic deviations (MAD), in degrees) as a function of the tilt direction.

The ANOVA also revealed an interaction between *head position *and *angle size *(F_(2,76) _= 10.549; *p *< .00001 (see Figure 3)). An orthogonal contrast analysis showed that the effect of angle size was significant for the head-tilted conditions (t_(38) _= 3.87, *p *< 0.001, for "left-tilt", t_(38) _= 2.00, *p *= 0.025, for "right-tilt") but not for the "head vertical" condition (t_(38) _= 1.24; *p *= 0.11).

When adjustments were made with a small angle size (0.95°), they tended to deviate away from the direction of the head tilt. In contrast, a large angle size (18.92°) led to deviations in the direction of the head tilt. None of the other single effects or interactions was significant (*p *> 0.19 for the effect of angle size and Fs < 1 for all the other factors and interactions).

In order to clarify the additive effects of gender and angle size on the SVV, we further analyzed the "head-tilt indexes". To that end, the mean obtained in the baseline condition (i.e. the head aligned with the vertical) was subtracted from the mean computed for the left- or right-tilted head condition, in order to isolate a "pure" head-tilt effect. Moreover, each mean was expressed with respect to the head axis (30°), in order to rule out the normal, reverse deviation caused by head-tilt to the left or to the right, respectively. Thus, an accurate gravitational judgment would be equal to 30°. In contrast, a value below 30° would reflect an A-effect and a value above 30° would reflect an E-effect.

**Figure 4:**
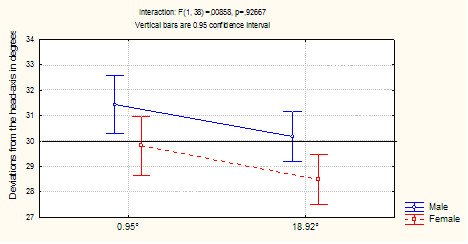
**Deviations from the head axis (in degrees), as a function of gender and line angle size**. The vertical bars represent the 95% confidence interval.

The standard assumptions for performing ANOVA were observed, i.e. a normal data distribution (checked with a Kolmogorov-Smirnov test) and a homogeneous variance-covariance matrix (checked with a Box's M test). We performed a two (*gender*) × two (*head position*) × two (*angle size*) factor ANOVA with repeated measures on the two latter factors. This analysis revealed a significant effect of angle size (F_(1,38) _= 12.654; *p *= 0.001). When adjustments were made with the small angle size (0.95°), the errors tended to be in the opposite direction to that of the head tilt (M = 30.630°; an E-effect). In contrast, a large angle size (18.92°) led to a slight deviation in the direction of the head (M = 29.340°; an A-effect). The effect of gender was also significant (F_(1,38) _= 6.307; *p *= 0.016). Men generally showed an E-effect (M = 30.809°), whereas women generally showed an A-effect (M = 29.160°). The two factors *gender *and *angle size *had additive effects, since the interaction between them was not statistically significant (F < 1; see Figure 4).

There was no significant effect of *head tilt *(F < 1). The SVV with the head tilted to the left (M = 29.871°) did not differ from that obtained with the head tilted to the right (M = 30.098°). The interaction between *gender *and *head tilt *failed to achieve statistical significance (F_(1,38) _= 2.667; MSE = 5.4; *p *= 0.11). All other interactions were non-significant (F < 1).

## Discussion

Our study results showed moderate SVV effects with a 30° head tilt. However, our experiment notably revealed that two methodological factors (the participant's gender and the angle size of the stimulus) had a statistically significant effect on the SVV in general and on the head-tilt effect in particular. Our second data analysis revealed that gender and angle size had additive effects on the deviation of the estimated SVV. The women tended to deviate their SVV in the direction of the head-tilt, whereas the opposite effect was found in men. Moreover, the head-tilt effect was modulated by the stimulus angle size. A large stimulus angle size led to deviations in the direction of the head-tilt, whereas a small angle size gave the opposite effect.

We suggest that these methodological factors could be partly involved in some of the differences found in the literature concerning the effect of head-tilt on the SVV. For instance, an experiment in which the SVV is tested with a large stimulus angle size in a predominantly women group (e.g. [23]) would be more likely to give rise to an A-effect (i.e. under-compensation of head-tilt; deviation of the SVV towards the head). In contrast, an experiment conducted with presentation of small angle size in a group of men would be more likely to an E-effect (i.e. overcompensation of head-tilt; deviation away from the head) on average. In the two other cases (women with a small angle size and men with a large stimulus angle size), the mean SVV may be close to the true physical vertical and would not display systematic deviation.

The difference between men and women observed here contrasts with the lack of an effect in previous studies [36,39]. However, our results fit well with Tremblay's assertion [32] that gender differences in the perception of spatial orientation are linked to anatomical differences in the vestibular apparatus. As mentioned above, Sato et al. [33] found that the otoliths and superior semicircular canals are larger in men than in women. According to Tremblay [32], this physiological difference may mean that women are poorly sensitive to vestibular information. The higher weighting given to vestibular signals could explain the men's propensity for an E-effect via overcompensation of head tilt (see Figure 4).

Our results on the effect of angle size agree rather well with the previous work by Wade [26], i.e. an increase in the E-effect with a small-sized stimulus angle. Although the gender of the ten participants was not stated in Wade's paper, an assessment of solely male participants would explain the lack of an A-effect in the 28.52° angle size condition.

Although the overall effects remain difficult to explain, some degree of speculation is justified. The effect of the angle size factor could be studied by rigorous investigation of eye movements and restriction of the allowed adjustment time (to avoid scanning movements, for example). Indeed, head tilt induces a vestibulo-ocular reflex (ocular countertorsion) that might lead to a visual E-effect (i.e. overestimation of head tilt) if the system does not take into account the slight countertorsion of the retinal meridians (usually about 10% of the head-tilt). Some researchers have considered that countertorsion is the predominant factor in SVV deviations [40] and SVV variability [41], although other researchers failed to find a link between eye torsion and the SVV [25].

Moreover, angle size differences could trigger additional eye movements. Although the 0.95° stimulus may be foveated, saccades and volitional eye movements are certainly required to fully view an 18.9°-sized line. Even though it is now clear that several regions of the human cortex contain orientation-selective neurons (V1, V2, V3, V3A and V4 in particular; for a review, see [42]), it is still not known where in the brain vestibular signals are integrated with visual signals (in order to convert the representation of visual stimulus from a retinal frame of reference into a world-centered frame of reference [43]). In humans, Corbett et al. [44] used event-related potential experiments to show that in the presence of a tilted frame, the SVV involved later, post-perceptual information processing. Moreover, Vandenberghe et al.'s PET study [45] showed that the brain areas involved in orientation discrimination are activated differently by a main stimulus and by the addition of a peripheral stimulus. The two stimuli used in our present research, a centrally viewed bar (0.95°) and a more peripherally viewed bar (18.95°), may have activated different cortical areas and thus generated different behavioral results (i.e. different SVV deviations).

Our study had several limitations. Firstly, the deviations of the SVV found with a 30° head tilt were quite moderate. One can legitimately postulate that factors not taken into account here (such as handedness) may have contributed to the great between-subject variability and thus the small overall deviation induced by head tilt. Thus, it would be useful to control for this factor and increase the number of participants. The second limitation relates to the fact that only a moderate head tilt (30°) was studied here; it would be interesting to investigate several larger tilt angles. Thirdly, it is difficult to interpret our data from a physiological point of view. However, as suggested by one of the reviewers of this article, it would be useful to check the validity of our findings by estimating both the subjective visual and haptic verticals in the same participants. This would enable us to determine whether the effects of gender and angle size observed here are linked to the central processing of visual information or, in contrast, otolithic information (see [8]).

## Conclusion

In summary, our study revealed the impact of two factors on evaluation of the SVV: the angle size of the stimulus and the participant's gender. Given the growing body of research that uses the SVV paradigm as an indicator of spatial orientation (particularly in disease settings), this methodological issue is important and may explain (at least in part) the discrepancies found in the literature on the head-tilt effect.

## Authors' contributions

ML conceived the design of the study, helped in the statistical analysis and wrote the manuscript. MN carried out the experiment, performed the statistical analysis and helped to draft the manuscript. VT carried out the experiment, performed the statistical analysis and helped to review the manuscript. EG helped to design the study and helped to draft the manuscript. All authors read and approved the final manuscript.

## References

[B1] Bauermeister M Werner H Wapner S The effect of body tilt on tactual-kinesthetic perception of verticality Am J Psychol 1964 77 451 456 10.2307/1421016 14198668

[B2] Guerraz M Luyat M Poquin D Ohlmann T The role of neck afferents in subjective orientation in the visual and tactile sensory modalities Acta Otolaryngol 2000 120 6 735 738 10.1080/000164800750000261 11099150

[B3] Guardia D Cottencin O Thomas P Dodin V Luyat M Spatial orientation constancy is impaired in anorexia nervosa Psychiatry Res 2012 195 1-2 56 59 10.1016/j.psychres.2011.08.003 21872340

[B4] Lejeune L Thouvarecq R Anderson DJ Caston J Jouen F Kinaesthetic and visual perceptions of orientations Perception 2009 38 7 988 1001 10.1068/p6132 19764301

[B5] Luyat M Gentaz E Corte TR Guerraz M Reference frames and haptic perception of orientation: body and head tilt effects on the oblique effect Percept Psychophys 2001 63 3 541 554 10.3758/BF03194419 11414140

[B6] Luyat M Verticale subjective versus verticale posturale: une note sur l'étude de la perception de la verticale (Subjective vertical vs postural vertical: a note on the perception of verticality L'Année Psychologique 1997 97 433 44722423300

[B7] Wright WG Glasauer S Haptic subjective vertical shows context dependence: task and vision play a role during dynamic tilt stimulation Ann N Y Acad Sci 2003 1004 531 535 10.1196/annals.1303.069 14662511

[B8] Schuler JR Bockisch CJ Straumann D Tarnutzer AA Precision and accuracy of the subjective haptic vertical in the roll plane BMC Neurosci 2010 11 83 10.1186/1471-2202-11-83 20630097 PMC2912915

[B9] Funk J Finke K Muller HJ Preger R Kerkhoff G Systematic biases in the tactile perception of the subjective vertical in patients with unilateral neglect and the influence of upright vs. supine posture Neuropsychologia 2010 48 1 298 308 10.1016/j.neuropsychologia.2009.09.018 19782092

[B10] Schwarz U Neuro-ophthalmology: a brief Vademecum Eur J Radiol 2004 49 1 31 63 14975493 10.1016/j.ejrad.2003.09.006

[B11] Guldin WO Grüsser O-J Is there a vestibular cortex? Trends Neurosci 1998 21 6 254 259 10.1016/S0166-2236(97)01211-3 9641538

[B12] Fasold O von Brevern M Kuhberg M Ploner CJ Villringer A Lempert T Wenzel R Human vestibular cortex as identified with caloric stimulation in functional magnetic resonance imaging NeuroImage 2002 17 3 1384 1393 10.1006/nimg.2002.1241 12414278

[B13] Suzuki M Kitano H Ito R Kitanishi T Yazawa Y Ogawa T Shiino A Kitajima K Cortical and subcortical vestibular response to caloric stimulation detected by functional magnetic resonance imaging Cognitive Brain Res 2001 12 3 441 449 10.1016/S0926-6410(01)00080-5 11689304

[B14] Barra J Marquer A Joassin R Reymond C Metge L Chauvineau V Perennou D Humans use internal models to construct and update a sense of verticality Brain 2010 133 Pt 12 3552 3563 21097492 10.1093/brain/awq311

[B15] Bergenius J Tribukait A Brantberg K The subjective horizontal at different angles of roll-tilt in patients with unilateral vestibular impairment Brain Res Bull 1996 40 5-6 385 390 discussion 390-381 10.1016/0361-9230(96)00131-1 8886363

[B16] Anastasopoulos D Bronstein AM A case of thalamic syndrome: somatosensory influences on visual orientation J Neurol Neurosurg Psychiatry 1999 67 3 390 394 10.1136/jnnp.67.3.390 10449566 PMC1736522

[B17] Karnath HO Dieterich M Spatial neglect-a vestibular disorder? Brain 2006 129 Pt 2 293 305 16371409 10.1093/brain/awh698

[B18] Brandt T Dieterich M Danek A Vestibular cortex lesions affect the perception of verticality Ann Neurol 1994 35 403 412 10.1002/ana.410350406 8154866

[B19] Kerkhoff G Zoelch C Disorders of visual orientation in the frontal plane in patients with visual neglect following right or left parietal lesions Exp Brain Res 1998 122 1 108 120 10.1007/s002210050497 9772118

[B20] Funk J Finke K Muller HJ Utz KS Kerkhoff G Effects of lateral head inclination on multimodal spatial orientation judgments in neglect: evidence for impaired spatial orientation constancy Neuropsychologia 2010 48 6 1616 1627 10.1016/j.neuropsychologia.2010.01.029 20138897

[B21] Funk J Finke K Muller HJ Utz KS Kerkhoff G Visual context modulates the subjective vertical in neglect: evidence for an increased rod-and-frame-effect Neuroscience 2011 173 124 134 21073929 10.1016/j.neuroscience.2010.10.067

[B22] Guerraz M Poquin D Luyat M Ohlmann T Head orientation involvement in assessment of the subjective vertical during whole body tilt Percept Mot Skills 1998 87 2 643 648 10.2466/pms.1998.87.2.643 9842617

[B23] Luyat M Gentaz E Body tilt effect on the reproduction of orientations: studies on the visual oblique effect and subjective orientations J Exp Psychol Hum Percept Perform 2002 28 4 1002 1011 12190248

[B24] Trousselard M Cian C Nougier V Pla S Raphel C Contribution of somesthetic cues to the perception of body orientation and subjective visual vertical Percept Psychophys 2003 65 8 1179 1187 10.3758/BF03194843 14710953

[B25] Mast FW Does the world rock when the eyes roll? Allocentric orientation representation, ocular counterroll, and the subjective vertical Swiss J Psychol 2000 59 89 101 10.1024//1421-0185.59.2.89

[B26] Wade NJ The effect of stimulus line variations on visual orientation with head upright and tilted Aust J Psychol 1969 21 2 177 185 10.1080/00049536908257782

[B27] Tribukait A Bergenius J Brantberg K The subjective visual horizontal for different body tilts in the roll plane: characterization of normal subjects Brain Res Bull 1996 40 (5 6):375-381 10.1016/0361-9230(96)00130-x 8886362

[B28] De Graaf B Bekkering H Erasmus C Bles W Influence of visual, vestibular, cervical, and somatosensory tilt information on ocular rotation and perception of the horizontal J Vest Res 1992 2 15 30 1342382

[B29] Kaptein RG Van Gisbergen JA Interpretation of a discontinuity in the sense of verticality at large body tilt J Neurophysiol 2004 91 5 2205 2214 10.1152/jn.00804.2003 14668294

[B30] Van Beuzekom AD Van Gisbergen JA Properties of the internal representation of gravity inferred from spatial-direction and body-tilt estimates J Neurophysiol 2000 84 1 11 27 10899179 10.1152/jn.2000.84.1.11/F

[B31] Tremblay L Elliott D Sex differences in judging self-orientation: the morphological horizon and body pitch BMC Neurosci 2007 8 6 10.1186/1471-2202-8-6 17207289 PMC1779793

[B32] Tremblay L Elliott D Starkes JL Gender differences in perception of self-orientation: software or hardware? Perception 2004 33 3 329 337 10.1068/p5209 15176617

[B33] Sato H Sando I Takahashi H Computer-aided three-dimensional measurement of the human vestibular apparatus Otolaryngol Head Neck Surg 1992 107 3 405 409 1408226 10.1177/019459989210700311

[B34] Asch SE Witkin HA Studies in space orientation: II. Perception of the upright with displaced visual field and body tilted J Exp Psychol 1948 38 455 477 18874603 10.1037/h0054121

[B35] Robert M Ohlmann T Water-level representation by men and women as a function of rod-and-frame proficiency and visual and postural information Perception 1994 23 1321 1333 10.1068/p231321 7761243

[B36] Barnett-Cowan M Dyde RT Thompson C Harris LR Multisensory determinants of orientation perception: task-specific sex differences Eur J Neurosci 2010 31 10 1899 1907 10.1111/j.1460-9568.2010.07199.x 20584195

[B37] Bell EC Willson MC Wilman AH Dave S Silverstone PH Males and females differ in brain activation during cognitive tasks NeuroImage 2006 30 2 529 538 10.1016/j.neuroimage.2005.09.049 16260156

[B38] Viaud-Delmon I Ivanenko YP Berthoz A Jouvent R Sex, lies and virtual reality Nat Neurosci 1998 1 1 15 16 10.1038/215 10195102

[B39] Groberg DH Dustman RE Beck EC The effect of body and head tilt in the perception of vertical: Comparison of body and head tilt with left and right handed, male and female subjects Neuropsychologia 1969 7 89 100 10.1016/0028-3932(69)90048-7

[B40] Wade S Curthoys I The effect of ocular torsional position on perception of the roll-tilt of visual stimuli Vis Res 1997 37 8 1071 1078 10.1016/S0042-6989(96)00252-0 9196725

[B41] Tarnutzer AA Bockisch CJ Straumann D Head roll dependent variability of subjective visual vertical and ocular counterroll Exp Brain Res 2009 195 4 621 626 10.1007/s00221-009-1823-4 19415246

[B42] Orban GA Vogels R The neuronal machinery involved in successive orientation discrimination Prog Neurobiol 1998 55 2 117 147 10.1016/S0301-0082(98)00010-0 9618746

[B43] Daddaoua N Dicke PW Thier P The subjective visual vertical in a nonhuman primate J Vis 2008 8 3 19 11 18 18484825 10.1167/8.3.19

[B44] Corbett JE Enns JT Handy TC Electrophysiological evidence for a post-perceptual influence of global visual context on perceived orientation Brain Res 2009 1292 82 92 19632209 10.1016/j.brainres.2009.07.038

[B45] Vandenberghe R Dupont P De Bruyn B Bormans G Michiels J Mortelmans L Orban GA The influence of stimulus location on the brain activation pattern in detection and orientation discrimination. A PET study of visual attention Brain 1996 119 Pt 4 1263 1276 8813289 10.1093/brain/119.4.1263

